# Framework for the assessment of PEMS (Portable Emissions Measurement Systems) uncertainty

**DOI:** 10.1016/j.envres.2018.06.012

**Published:** 2018-10

**Authors:** Barouch Giechaskiel, Michael Clairotte, Victor Valverde-Morales, Pierre Bonnel, Zlatko Kregar, Vicente Franco, Panagiota Dilara

**Affiliations:** a European Commission, Joint Research Centre, via E. Fermi 2749, 21027 Ispra, Italy; b European Commission, Directorate-General for Environment, Avenue de Beaulieu 9 (BU-9 04/091), 1160 Brussels, Belgium; c European Commission, Directorate-General for Internal Market, Industry, Entrepreneurship and SMEs, BREY 10/30, B-1049 Brussels, Belgium

**Keywords:** Air pollution, Vehicle emissions, Real driving emissions (RDE), Portable emissions measurement systems (PEMS), Conformity factor, Nitrogen oxides (NOx)

## Abstract

European regulation 2016/427 (the first package of the so-called Real-Driving Emissions (RDE) regulation) introduced on-road testing with Portable Emissions Measurement Systems (PEMS) to complement the chassis dynamometer laboratory (Type I) test for the type approval of light-duty vehicles in the European Union since September 2017. The Not-To-Exceed (NTE) limit for a pollutant is the Type I test limit multiplied by a conformity factor that includes a margin for the additional measurement uncertainty of PEMS relative to standard laboratory equipment. The variability of measured results related to RDE trip design, vehicle operating conditions, and data evaluation remain outside of the uncertainty margin. The margins have to be reviewed annually (recital 10 of regulation 2016/646). This paper lays out the framework used for the first review of the NO_x_ margin, which is also applicable to future margin reviews. Based on experimental data received from the stakeholders of the RDE technical working group in 2017, two NO_x_ margin scenarios of 0.24–0.43 were calculated, accounting for different assumptions of possible zero drift behaviour of the PEMS during the tests. The reduced uncertainty margin compared to the one foreseen for 2020 (0.5) reflects the technical improvement of PEMS over the past few years.

## Highlights

• Portable Emissions Measurement Systems (PEMS) are used to measure Real-Driving Emissions (RDE) of vehicles.
• The uncertainty of PEMS was evaluated based on experimental data and technical requirements prescribed in the European regulation.
• The 2017 uncertainty estimation was 43%.

## Graphical abstract

**Figure:**
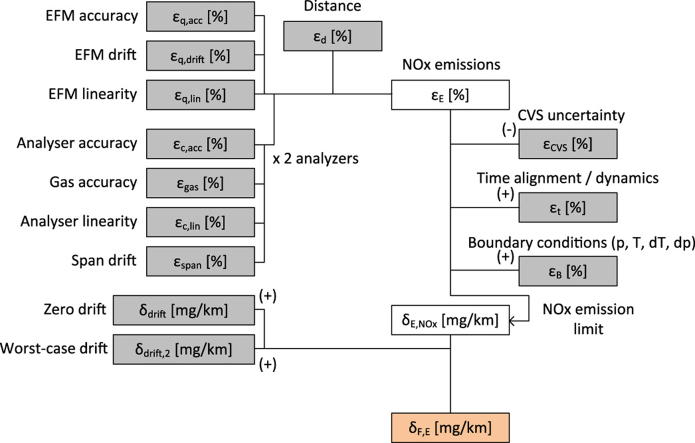

## 1 Introduction

Nitrogen oxides (NO_x_) (especially nitrogen dioxide, NO_2_) are major air pollutants due to their role as precursors of smog (WHO, 2013). They react with water to produce nitric acid, which is irritating to the eyes and respiratory tract. They can cause inflammation, respiratory diseases, decreased lung function, and increased reactions to allergens. Moreover, they contribute to the formation of secondary particulate matter (PM) and ground-level ozone. Exposure to NO_2_ in ambient air led to an estimated 78.000 premature deaths across the population of 41 European countries in 2014 (EEA, 2017).

Road traffic contributes significantly to urban air pollution. The annual limit value for NO_2_ continues to be exceeded across Europe, with around 10% of all the reporting stations recording concentrations above the standard in 2015 in a total of 22 of the 28 European Union (EU) countries and three other reporting countries. Almost 90% of all concentrations above this limit value were observed at traffic stations (EEA, 2017). A recent study demonstrated that vehicle exhaust has a far greater impact on concentrations of NO_2_ than PM (Harrison and Beddows, 2017). Although PM concentrations have been declining the last decade due to the use of diesel particle filters, this is not true for NO_x_ (Harrison and Beddows, 2017).

NO_x_ emissions from vehicles in Europe are regulated through the Euro standards, which were first introduced in the 1990s. Since that time, the allowable limits have been progressively tightened. At the same time, the gap between NO_x_ measurements in laboratory tests and real-driving emissions (RDE) has been increasing for diesel vehicles. For Euro 3 diesel vehicles (limit 500 mg/km), the difference was a factor of 2, for Euro 5 (limit 180 mg/km) it increased to 3–4 (Baldino et al., 2017, Transport and Environment, 2016), whereas the first Euro 6 cars introduced between 2012 and 2015 (limit 80 mg/km) on the market were typically emitting 3–7 (Transport and Environment, 2016, Franco et al., 2014) times more NO_x_ than allowed. Similarly, a limited number of studies showed that Euro 5 diesel light commercial vehicles, like vans, (limit 280 mg/km) had 5–6 times higher emissions on the road (Kadijk et al., 2016). The main reasons for such discrepancies are: a) the lack of representativeness of the type approval procedure (specifically the NEDC test, a chassis dynamometer driving cycle with an artificial driving profile), and b) the use of defeat devices (i.e., illegitimate emission control strategies, such as “cycle detection) or ”thermal window”, which reduce the effectiveness of the emission control system under driving conditions not covered by the type approval procedure and which were at the centre of the diesel emissions scandal in Europe (Degraeuwe and Weiss, 2017).

The gap between official laboratory results and the actual on-road emissions led to a revision of the type approval requirements in the EU. To that end, a technical working group on RDE was set up in 2011. The work of the RDE group produced several pieces of legislation: Commission Regulation (EU) 2016/427 (first regulatory act of the RDE regulation, RDE1) (RDE1, 2016) introduced on-road testing with Portable Emissions Measurement Systems (PEMS) to complement the laboratory Type I test for the type approval of light-duty vehicles in EU. Subsequently, Commission Regulation (EU) 2016/646 (RDE2, 2016) introduced the Not-To-Exceed (NTE) limits which are the emission limits for the laboratory Type I test multiplied by a so-called conformity factor that takes into account the measurement uncertainty of the PEMS. Both regulations were consolidated in the World Harmonised Light Duty test Procedure (WLTP) Commission Regulation (EU) 2017/1151 (WLTP, 2017) and further developed by Commission Regulation (EU) 2017/1154 (RDE3, 2017), which also introduced a RDE conformity factor for the on-road test of Particle Number (PN) emissions. The fourth part of the RDE regulation introduced on-road emissions testing as part of in-service conformity checks (RDE4, 2018).

The objective of this paper is to outline the framework for the annual systematic reviews and revisions of PEMS measurement uncertainty, which determines the conformity factor. This framework can be useful for researchers conducting tests with PEMS to characterise the uncertainty of their measurements.

## 2 Background

According to the RDE regulations, a first-step, temporary conformity factor of 2.1 for NO_x_ tailpipe emissions may apply from September 2017 upon the request of the manufacturer. From January 2020, a second-step conformity factor (currently 1.5) will apply for all new type approvals. This conformity factor allows a measurement margin (currently 0.5 or 50%) solely to account for the additional uncertainty of PEMS relative to standard laboratory equipment (recital 10 of Commission Regulation 2016/646, (RDE2, 2016)). The recitals in the RDE regulations require the Commission to review annually the appropriate level of the final conformity factor in light of technical progress, a task that was undertaken by the European Commission's Joint Research Centre (JRC).

### 2.1 Rationale for the definition of the NO_x_ conformity factor

To obtain a quantitative estimate of the measurement uncertainty, JRC conducted an assessment of PEMS and laboratory equipment in 2015 (Weiss et al., 2015) based on the technical performance requirements laid down for PEMS and laboratory equipment in the RDE Commission Regulation 2016/427 (RDE1, 2016) and in UNECE regulation 83 (2015), respectively. This assessment was complemented by a scenario analysis based on emission measurements conducted with 4 vehicles with engine displacements ranging from 1.2 to 3.0 litres. The results that were presented to the RDE working group in October 2015 suggested that PEMS test might be subject to up to 30% higher measurement uncertainty than the laboratory test (i.e., an uncertainty margin of 0.3), broken down as follows: (i) 10% (margin 0.1) additional uncertainty resulting from the performance requirements for PEMS analysers, exhaust flow meter, and the vehicle speed signals. (ii) 20% (margin 0.2) additional uncertainty resulting from possible analyser drift affecting the second-by-second measurement of NO_x_ concentrations during an on-road test. Analyser drift is virtually negligible in the laboratory, as the NO_x_ concentration (and that of other pollutants) in the sampling bags is determined once at the end of a test, immediately after a calibration of the analyser, rather than over longer periods (circa 2 h) on a second-by-second basis as it is done with PEMS.

This first assessment of the PEMS uncertainty margin for NO_x_ was however limited to vehicles with 1.2–3.0 litres engines, and it assumed a gradual (linear) drift over the test. This meant that assuming a worst-case scenario for the drift (maximum allowable drift occurring from the beginning of the test) and taking into account the increased effect of drift (expressed in mg/km) for engines with displacement above 3.0 litres, the uncertainty margins could, in some cases, exceed those quantified initially by the JRC. Taking these observations into account, the currently established NO_x_ margin of 0.5 can be regarded as a conservative estimate of the additional uncertainty of NO_x_ emissions measured with PEMS for a very broad range of engine displacements. An annual review clause was introduced in the legislation in order to allow for further improvements and analysis.

### 2.2 Review activities and amendments implemented in 2016

In 2016, the European Commission organized two stakeholder meetings dedicated on the issue of uncertainty of PEMS measurements. In these meetings, PEMS manufacturers expressed their support to reduce the maximum allowable zero drift for NO_x_ analysers by 50% through a revision of Table 2 of Appendix 1 of Commission Regulation 2016/427 (RDE1, 2016). This table specified that the zero and span drift over a test had to be within 5 ppm and 2% of the reading, respectively. The provision used to apply individually to NO_2_ and NO/NO_x_ measurements. As NO_x_ is calculated as the sum of the measured NO_2_ and NO concentrations, the effective allowable NO_x_ zero drift was thus 10 ppm. The revised provisions in Commission Regulation 2017/1154 (RDE3, 2017) clarified that NO_x_ concentrations are to be determined within a zero drift of 5 ppm. The amendment thereby lowered the permissible drift for NO_x_ measurements by 50% compared to the original requirements in Commission Regulation 2016/427 (RDE1, 2016) (in line with the recommendations of PEMS manufacturers), which in turn provided the scope for revising the PEMS uncertainty margin for NO_x_.

### 2.3 Review activities in 2017

The RDE regulation requires the European Commission to “keep under annual review the appropriate level of the final conformity factor in light of technical progress”. To this end, “appropriate level” should be understood as the level of conformity factor that is justified by the additional measurement uncertainty of PEMS which comply with the performance requirements of the RDE regulation, relative to the laboratory equipment. The term “technical progress” should be understood as improved PEMS measurement performance achieved in real-world use, and/or prescribed by more stringent regulatory RDE requirements with regard to measurement equipment performance criteria.

The review of the PEMS measurement uncertainty therefore focused on quantifiable error sources resulting from the technical performance requirements defined in the RDE regulation (e.g. for NO_x_ analyser drift, accuracy of analysers and exhaust flow meters). The variability of measured results related to the RDE trip design, vehicle operating conditions, and ex-post data evaluation remained outside of the uncertainty margin and thus outside of the scope of this paper.

The 2017 review focused on the definition of the framework for the assessment of the measurement uncertainty. Experimental data from the laboratory and from on-road measurements identified areas that needed attention and further input (Giechaskiel et al., 2018).

## 3 Theoretical background

### 3.1 Overview of technical requirements for PEMS

A Portable Emission Measurement System (PEMS) generally consists of 1) pollutant analysers, 2) an exhaust flow measurement (EFM) device, 3) a Global Positioning System (GPS), 4) auxiliary sensors (ambient temperature and pressure, etc.), and 5) a power supply. The distance-specific emissions are calculated based on the signals from the analyser, the exhaust flow meter and the positioning system (with distance being derived from an instantaneous velocity signal). Commission Regulation 2016/427 (RDE1, 2016) describes the technical requirements for PEMS measuring devices. These requirements result in a theoretical measurement uncertainty (more details in the following section).

The most important requirements prescribed in the RDE regulation for the analysers and the EFM that have direct impact on the PEMS measurement uncertainty are:

Accuracy (at a specific concentration). For NO_x_ (concentration measured by the analyser) and EFMs (flow rate measured) it is set at 2% of the reading.

Non-linearity (differences at low – high concentrations). The permissible variability expressed as standard error of estimate (SEE) is set at 1% for NO_x_ and at 2% of reading for EFMs.

Drift over time for zero and maximum concentration (span). For NO_x_, the permissible zero and span drift is set at 5 ppm and 2% of reading, respectively. For EFMs it is set at 1% of reading.

A comparison of the PEMS with the laboratory equipment on a chassis dynamometer can be done in order to check of the functionality of the complete PEMS once it is fully installed in the vehicle. In the context of RDE measurements, this procedure is called as “validation of PEMS” and is not meant to compare the respective measurement performance of the laboratory and the PEMS test principles. Such a validation test only ensures that the PEMS is correctly installed and functioning when its emissions over a WLTC are found to be within a reasonable range of the ones given by the constant-volume sampling (CVS) system of the laboratory. The permissible tolerances of this validation are given in the RDE regulation. For example, for NOx, the limits are set to ± 15% of the result or 15 mg/km, whichever is larger.

### 3.2 Uncertainty framework

The emissions of NO_x_ in the RDE regulation, *E*, are calculated from the following equation:

$$E=\frac{\sum uc_{i}q_{i}}{d}$$

where:

**Table:** 

*u*	is the ratio of the density of NO_x_ and the overall density of the exhaust (constant for a fuel),
$c_{i}$	is the NO_x_ instantaneous measured concentration in the exhaust at time *i* [ppm],
$q_{i}$	is the measured instantaneous exhaust mass flow rate at time *i* [kg/s],
*d*	is the distance of the test [km].

For the estimation of the NO_x_ emissions uncertainty ($\varepsilon_{Ε}$) (in %), the error propagation rule for multiplication and division was used (Farrance and Frenkel, 2012). This assumes errors are random and uncorrelated to each other, which is a valid assumption for a PEMS setup (e.g. the error of the GPS is not correlated to that of the NO_x_ analyser). The constant *u* does not contribute to the relative uncertainty $\varepsilon_{E}$:

$$\varepsilon_{E}=\sqrt{\varepsilon_{q}^{2}\;+\;\varepsilon_{c}^{2}\;+\;\varepsilon_{d}^{2}}$$

where

**Table:** 

$\varepsilon_{q}$	is the relative uncertainty of the exhaust mass flow rate [%],
$\varepsilon_{c}$	is the relative uncertainty of the NO_x_ concentration [%],
$\varepsilon_{d}$	is the relative uncertainty of the distance [%].

Note that this uncertainty equation assumes that the relative uncertainty remains constant for the different NO_x_ concentrations and exhaust flow rates. This assumption will be discussed in the “Discussion” section.

**Fig. 1:**
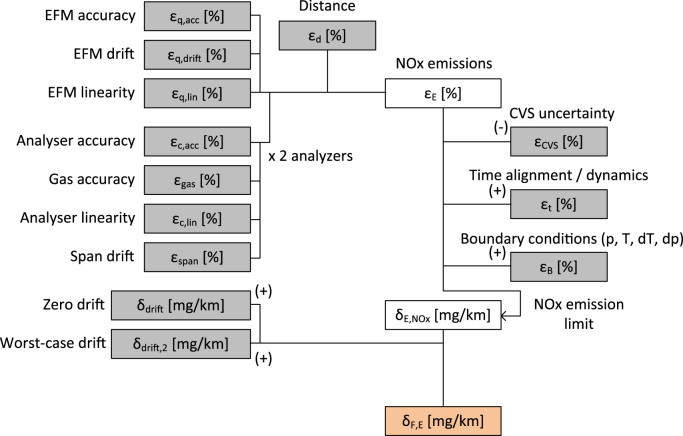
Uncertainties calculation scheme. Symbols (+) or (-) indicate that the errors are simply added or subtracted, without using the error propagation rule. “ε” refers to relative uncertainty and “δ” to absolute uncertainty.

In order to find the uncertainty of each component of the equation, the technical specifications in the RDE regulation and experimental data were taken into account. For example, the uncertainty of the analyser and the EFM is determined by the accuracy, linearity (standard error requirement), zero and span drift requirements (Fig. 1). Since regulation gives maximum uncertainties, $\varepsilon_{E}$ is the expanded uncertainty (i.e. a coverage factor of 2–3 is included already).

The uncertainty of the regulated laboratory CVS bag measurement (*ε*_*CVS*_) was subtracted from the overall uncertainty because the margin should cover only the additional uncertainty of PEMS equipment.

The zero drift of the analyser (*δ*_*drift*_) was analysed separately due to its significant effect on emissions at low concentrations. Note that this uncertainty is expressed in [mg/km] because the evaluation was in [mg/km]. The (absolute) uncertainty symbol is *δ*. Some additional uncertainties were also considered, such as time misalignment of signals (*ε*_*t*_), and the effect of test/environmental conditions, such as temperature, altitude, vibration (called boundary conditions in the regulation) on instrumentation accuracy (*ε*_*B*_). All these additional uncertainties were added to the uncertainty estimation in order to find the maximum uncertainty and to ensure that no RDE test would exceed the limit due to these additional uncertainties where the experience is limited (i.e. reduce Type I statistical error).

The final uncertainty *δ*_*F,E*_ [mg/km] for an emission level *L* [mg/km] is calculated as:

$$\delta_{F,E}=\left[\varepsilon_{E}+\varepsilon_{t}+\varepsilon_{B}-\varepsilon_{\mathrm{CVS}}\right]L+\delta_{\mathrm{drift}}$$

A simplified schematic of the uncertainties considered is shown in Fig. 1.

## 4 Materials and methods

The experimental data came from two main sources (Giechaskiel et al., 2018):

● Voluntary submissions under the “margins” sub-group of the RDE working group: The data came from 7 technical institutes, 2 instrument manufacturers, the Japan Automobile Manufacturers Association (JAMA) (data from one Original Equipment Manufacturer (OEM)), and the European Automobile Manufacturers' Association (ACEA) (data from 4 OEMs). They included 4 PEMS models/manufacturers; however, the majority of data were from 2 PEMS manufacturers. In total 162 tests were available for zero/span drift evaluation, as well as 162 cycles (>300 sub-cycles) from 101 vehicles for the validation tests.
● Data from the RDE reporting and monitoring exercise: RDE test results from 9 Member States were screened for data that could be used for the evaluation of the PEMS uncertainty margins (i.e., data that included a comparison with a reference laboratory system). From 415 RDE tests, 227 were usable for the drift evaluation. The data included also 66 “validations of PEMS” tests.

## 5 Results

The results of the theoretical analysis of the uncertainty using the technical requirements of RDE regulation or the experimental data of 2017 are summarized in Table 1. The following sections explain how the values were selected.

**Table 1:** (Values in bold were used in the further analysis. Uncertainty components that were not investigated are given in brackets.

**Name**	**Symbol**	**RDE**	**2017 review**	**Comments**
**EXHAUST FLOW METER (EFM**)				
EFM accuracy	*ε*_*q,acc*_	3%	**10%**	Fig. 2
EFM drift	*ε*_*q,drift*_	**2%**	neglig.	
EFM linearity	*ε*_*q,lin*_	**2%**	< 0.5%	
**GAS ANALYSERS**				
Analyser accuracy	*ε*_*c,acc*_	2%	**2%**	Fig. 3
Analyser linearity	*ε*_*c,lin*_	1%	**1%**	
Span drift	*ε*_*span*_	**2%**	≤ 5%	
Gas accuracy	*ε*_*gas*_	**2%**	[2%]	
**OTHER**				
Distance	*ε*_*d*_	**4%**	[4%]	
Dynamics	*ε*_*t*_	time aligned	**3%**	
Boundary conditions	*ε*_*Β*_	0%	**0%**	
**ZERO DRIFT**				
Analyser zero drift	*δ*_*drift*_	5 ppm	**10–15 mg/km**	Fig. 4
Worst-case drift	*δ*_*drift,2*_	–	**0–10 mg/km**	Fig. 4

### 5.1 Exhaust flow meter (EFM)

**Fig. 2:**
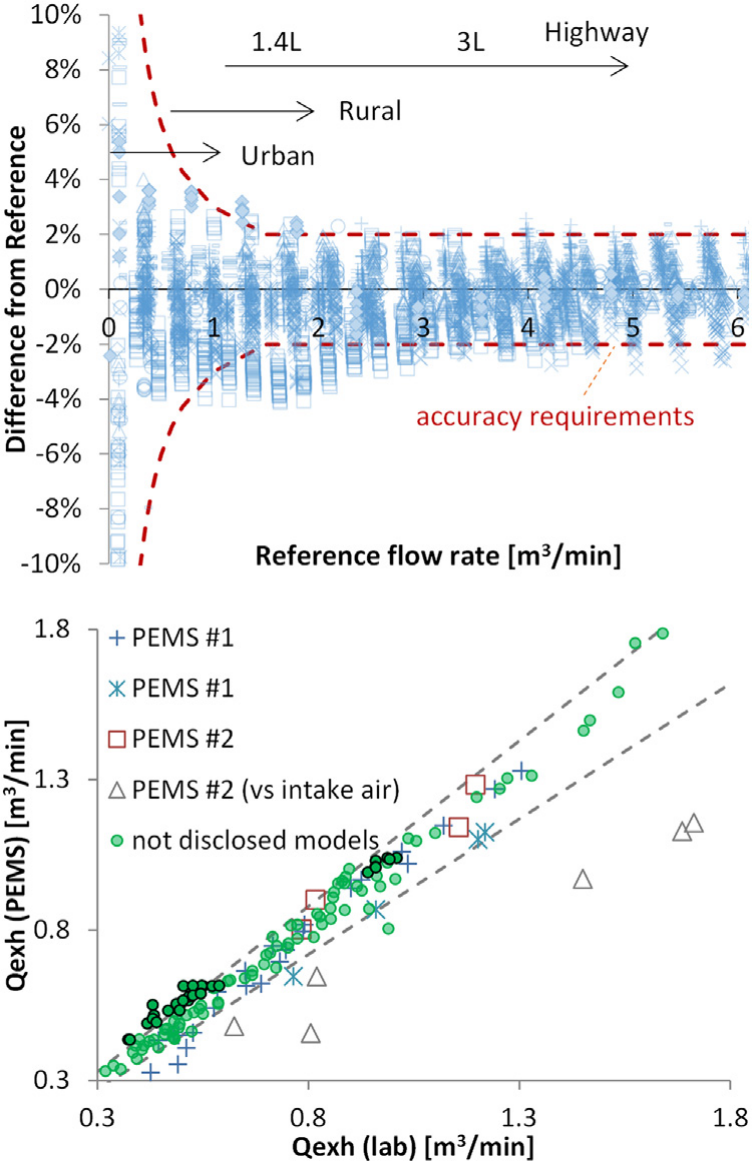
Upper panel: Checks of 44 light-duty EFMs against a traceable standard at the instrument manufacturer's site after 1 year of use (data from one instrument manufacturer). Arrows show typical exhaust flow rate ranges for various light-duty vehicles. Lower panel: Comparison of EFMs with other EFMs or CVS estimated flows. Dotted lines show ± 10% differences. Circles with black frame are from one stakeholder.

Fig. 2 (upper panel) shows calibration data of EFMs after one year of use. The EFM drift was negligible after one year of testing indicating that the drift during a test should also be negligible. Nevertheless, the maximum permissible value 2% was kept in the calculations of the measurement margin.

The EFM accuracy requirement as set in RDE regulation is 3% when compared with calibrated flow meters at laboratory conditions (constant mass flows of air at room temperature). As shown in Fig. 2 (upper panel), at low flow rates, such as idle, the uncertainty is high in relative terms (> 10%), but low in absolute values. For urban and rural conditions (flow rates <1 m^3^/min) the uncertainty is less than 4%. For higher flow rates, the uncertainty is < 3%. In all cases, the standard error of estimate (SEE), an indication of the linearity, is < 0.5% of the maximum value (compare to the 2% allowed by the regulation). Thus, at laboratory conditions the EFMs fulfil the requirements of the regulation.

Some investigations were also conducted under more realistic conditions. The received EFM evaluation data from the margins sub-group included comparisons with other EFMs, exhaust mass flow estimated by the CVS or engine intake air (as reported by the test vehicle´s on-board diagnostics, OBD) during test cycles. However, these methods have an uncertainty at the same level as the examined EFMs, and cannot be easily traceable. Thus, these results are only indications of EFM uncertainty. The differences between EFM evaluation data and the received data were within 10% (Fig. 2, lower panel), with the exception of all points of a specific stakeholder which indicated issues with the specific EFM (or the other comparison method). The EFM comparison with intake air, which does not take into account the fuel consumption, had, as expected, higher differences. At lower flow rates higher differences could also be seen. This could be due to EFM or reference instrument calibration errors and uncertainties at low flow rates.

In order to take into account the differences among EFMs in the market today and the difficulties checking them in practice, a conservative 10% uncertainty value was assumed.

### 5.2 NO_x_ analysers

**Fig. 3:**
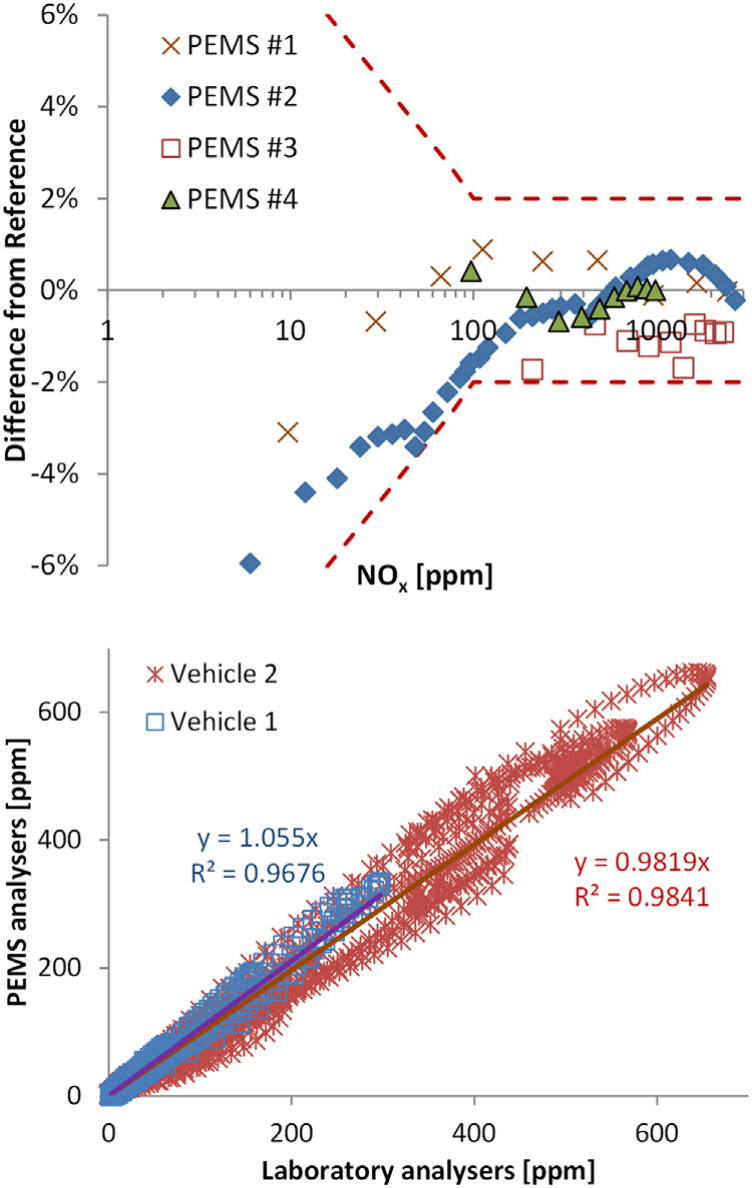
Upper panel: Examples of calibration data of NO analysers from 4 different PEMS manufacturers at different concentration levels. NO_2_ analysers are typically calibrated at lower range (< 1000 ppm). Lower panel: Correlation of second by second data of PEMS and laboratory grade analysers for two vehicles (NO_x_ emissions of vehicle 1: 10 mg/km, vehicle 2:200 mg/km).

The accuracy of the analysers, compared to reference instruments, should be 2% or better according to RDE regulation (from 10% of the maximum range up to the full measurement range). Data from the calibration certificates of the instruments (Fig. 3, upper panel) confirmed this accuracy from approximately 100 ppm levels for NO and 30 ppm for NO_2_. Additionally, a 1% non-linearity uncertainty for the gas analysers was considered (based on the SEE requirement of RDE).

The span drift, determined by comparing the analyser response to a reference span gas, was kept at 2% (as required in RDE) in the uncertainty calculations because higher values (even though they occurred in the received data) would result in an invalidation of the emissions tests. It should be noted that, from the 413 span drift tests, 12.1% were outside the permissible tolerance of 2%. The majority of them failed 1) in the laboratory indicating improper warmup time of the PEMS (span drift up to 5%), and 2) at ´extended´ conditions (i.e., more challenging test conditions allowed by the RDE regulations where special provisions apply). For example, an instrument had a drift of 11% for low ambient temperatures (this issue was later corrected). Applying a 2% span drift at different actual recordings (linearly from 0% at 0 ppm to +2% at the span concentration) showed that this 2% span drift has a < 2% effect on the final result. The maximum effect was seen when the emissions are coming from a few spikes close to the span level.

The uncertainty of the gas that is used for calibrations was assumed to be 2%, as required by the RDE regulations. Discussions with gas cylinder producers confirmed that this uncertainty is < 1.5%.

Based on the previous values, the analysers' uncertainty was estimated to be around 5%. The data that were received in 2017 confirm this value. For example, JAMA compared PEMS with laboratory-grade equipment (Fig. 3, lower panel). The mean differences ranged from around 2% (at final emission level of 200 mg/km, vehicle 2) to 6% (at final emission level of 10 mg/km, vehicle 1). Thus, at a level of 80 mg/km an uncertainty < 4% would correspond. However, higher differences can be observed when looking at second-by-second values, as shown by the scatterplot of the correlation.

### 5.3 Other

The distance uncertainty was kept at the 4% value assumed in the 2015 study, as no new analysis was conducted in 2017. This is the maximum allowed difference between two methods that have to be used to determine the distance in a RDE test (for example GPS vs. topographic map, ECU (Engine Control Unit), or an optical or micro-wave sensor). From the limited number of data received, the error was estimated around 1.5%.

The time alignment/dynamics uncertainty was kept at the 3% value from the 2015 study. Similar values (consistently <5%) were found from the limited number of real-time data received in 2017 (all were laboratory tests, no tests from the road). The evaluation was conducted by misaligning the EFM and gas analyser signals by ± 1 s and observing the resulting differences in calculated emissions. The misalignment is unlikely for commercial PEMS which define accurately the response time of the EFM and the analysers. Additionally, the misalignment can have a positive or negative effect on the result. Nevertheless, it was decided to add this uncertainty to take into account the dynamics of the EFM and the analysers (see for example Fig. 3, lower panel the high scatter).

The contribution of the environmental (boundary) conditions (−7 °C to +35 °C, up to 1300 m, and vibrations) was considered negligible, based on the results received from one instrument manufacturer. A second instrument manufacturer had issues at different temperature and pressure conditions, but this issue was later resolved with a simple hardware modification. Although the contribution is partly considered in the drift of the instruments, more detailed investigations are necessary. A CEN (Comité Européen de Normalisation) standard is under development to address the topic (CEN/TC 301, 2018).

In all cases, a 3% uncertainty of the CVS laboratory measurements was subtracted, as was also done in the 2015 analysis. As no new experimental data were received for the 2017 study, the CVS uncertainty was left at the 3% value from 2015.

### 5.4 Zero drift

The maximum allowed zero drift is 5 ppm (and no drift correction is allowed in the light-duty regulation). However, simply checking the zero level before and after the test is insufficient to characterise the influence of zero drift upon the uncertainty of the measurement, because it is not known how the drift, if any, built up over the test. Therefore, and to be able to assess this aspect, two scenarios of the zero drift were analysed:

A drift of 5 ppm happening immediately at the beginning of the test (t = 0 s) and remaining constant for the whole test. This scenario (representing an extreme assumption) is called “step zero drift”.

A drift increasing linearly from the beginning of the test and reaching 5 ppm at the end of the test. This scenario is called “linear zero drift”.

**Fig. 4:**
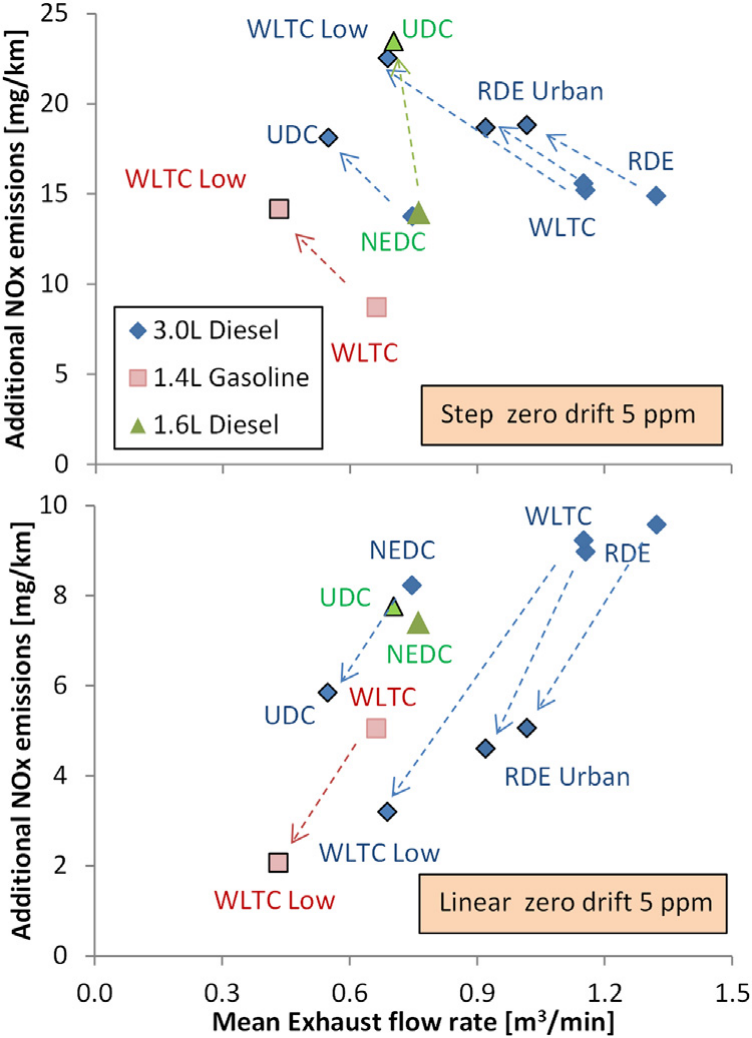
Effect of zero drift on final NO_x_ emissions for different cycles and vehicles in function of the mean exhaust flow of the specific test. Upper panel: 5 ppm step increase from t = 0 s. Lower panel: linear increase to reach 5 ppm at the end of the cycle. The arrows show the urban (low) part of the specific test cycles (which are in closed symbols). NEDC: New European Driving Cycle; WLTC: World harmonised Light-duty Test Cycle; RDE: Real Driving Emissions test; UDC: Urban Driving Cycle.

Although the drift can be negative, it only the positive drift effect was examined in order to avoid cases that a test would exceed the RDE limit due to drift. The drift effect was simulated for different driving cycles (New European Driving Cycle (NEDC), World harmonised Light-duty Test Cycle (WLTC), and Real Driving Emissions (RDE) cycles) and different typical vehicles technologies (diesel or gasoline) with different engine capacities ranging from 1.4 to 3.0 litres (Fig. 4) for the cases that the exhaust flow rates were available.

Fig. 4 (upper panel) shows the simulation results (additional NO_x_ emissions) for a “step zero drift”. The results are plotted in function of the mean exhaust flow rate. The arrows show the urban part of the respective cycles (e.g. Urban Driving Cycle (UDC) for NEDC, Low part for WLTC). The results show: (i) larger engines have higher mean exhaust flow rates over a given cycle (as expected) and thus the 5 ppm drift results in a larger change in NO_x_ emissions expressed in mg/km, (ii) for most cases, the maximum 5 ppm zero step drift translates to an increase of < 15 mg/km NO_x_ emissions over typical cycles (see NEDC, WLTC points), and (iii) a worst-case “step zero drift” for the largest engines and/or urban conditions (where the mean speed/distance is low) could translate to 20–25 mg/km NO_x_, i.e. an additional 5–10 mg/km of NO_x_ with respect to the typical < 15 mg/km NO_x_ contribution of the step zero drift (see difference NEDC-UDC and WLTC-WLTC Low).

Fig. 4 (lower panel) shows the simulation results for a linear increase of drift reaching 5 ppm at the end of the cycle (i.e., t = 1180 s for NEDC, t = 1800 s for WLTC or t > 5500 s for RDE). The results show: (i) for all cases, the linear 5 ppm drift translates to an increase of NOx emissions < 10 mg/km, (ii) a worst-case drift for the largest engines and/or urban conditions (where the mean speed and urban distance is low) was not observed. In other words, under this scenario, the drift effect at the urban part is negligible because the zero drift is very low at the beginning of the cycle.

In summary, for the worst-case “step zero drift” scenario, the zero drift has a contribution to the overall uncertainty of < 15 mg/km for RDE trips in most situations. An additional contribution to the uncertainty in the range of 5–10 mg/km is observed for the largest engines and/or urban conditions. For the “linear zero drift” scenario, the zero drift has a contribution to overall uncertainty of < 10 mg/km, and no extra uncertainty contribution is observed for the largest engines and/or urban conditions.

### 5.5 Combined uncertainty

**Fig. 5:**
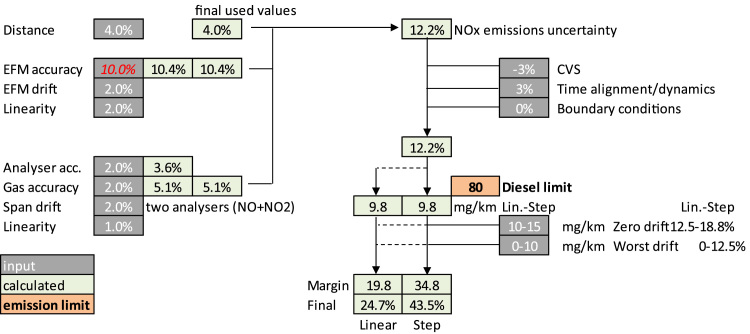
Combined RDE measurement uncertainty estimates for “step zero drift” and “linear zero drift” for NO_x_ emissions from diesel light-duty vehicles. In red italic values higher than the RDE requirements.

Fig. 5 plots the results of the margin analysis combining the uncertainty from this section using the methods described in Section 3 for diesel vehicles (limit 80 mg/km). The different assumptions for the zero drift determine the two scenarios leading to a combined uncertainty of 24% and 43% for linear and step increases respectively. This uncertainty is split to the proportional PEMS uncertainty (12% or 10 mg/km) and the constant zero drift uncertainty (10 mg/km to 25 mg/km). The above analysis shows that the bigger influence on the uncertainty arises from the zero drift: in our analysis, it depends on whether one assumes that it happens in a step change at the beginning of the test (worst case) or gradually during the test. Other zero drift cases which are also possible were not evaluated, because there is lack of data showing how zero drift typically occurs.

The analysis for gasoline vehicles or other emission levels will be discussed in Section 6.3.

It should be mentioned again here that this combined uncertainty calculates the additional uncertainty of the PEMS equipment compared to the laboratory systems. The uncertainty of a test result with PEMS is the same without subtracting the CVS uncertainty.

### 5.6 Validation tests

The comparison test between PEMS and chassis dynamometer laboratory (typically using the WLTC cycle) is called “validation”. The applicable rules for this comparison are laid down in Commission Regulation 2016/427 (RDE1, 2016). The framework presented in this paper can be applied to estimate the uncertainty of the “validation” test in the laboratory. The distance uncertainty is set to zero in this case, because the same distance is used for both the PEMS and the laboratory analysers. Typically, small drift, if any, is to be expected in the constant-temperature, vibration-free environment of a chassis dynamometer, thus the “linear zero drift” scenario should be valid for the laboratory conditions and was applied. Using a 10% uncertainty for the EFM, the final NO_x_ uncertainty is 16.9 mg/km, very close to the 15 mg/km limit imposed by the regulation. From the 178 validation tests available in the dataset, only 4% were outside the permissible tolerance of the regulation (15 mg/km or 15%, whatever is larger). This could indicate that the zero drift error in the chassis dynamometer is even smaller than 10 mg/km and/or the EFM error is smaller than < 10%. However, this uncertainty level for the PEMS is valid only in the laboratory, where the testing conditions are well-defined (constant temperature and pressure, absence of vibrations and shocks, etc.) and the test is shorter (only 30 min) and thus any influence on the EFM and analysers (e.g. drift) is small. The uncertainty of the on road trip covers additionally possible influence of test (boundary) conditions, additional drift effects due to the longer duration of the test (90 min vs. 30 min in the laboratory), uncertainty of the distance estimation, and possibly the different and wider working range of analysers and EFMs.

## 6 Discussion

This section will discuss the framework concept, the robustness of the uncertainty values that were used, possible ways of improving the framework concept and extending it to lower emission levels.

### 6.1 Comparison with other studies

The calculations scheme for the estimation of the PEMS measurement uncertainty, which translates to a conformity factor that is taken into account in the NTE limits, was based on statistical concepts of determining uncertainty (propagation of error) (Fig. 1). Although this is the first step in the European regulation, the uncertainty topic has been addressed already more than 10 years ago in the United States of America (USA). The measurement allowance in USA accounts for the PEMS measurement uncertainty and is added to the in-use limit for a Not-To-Exceed (NTE) combined limit. In USA all expected source of PEMS measurement errors were quantified using a series of controlled laboratory experiments and resulted in an error surfaces, which represent incremental errors of PEMS measurements as compared to laboratory reference measurements (Feist et al., 2009). Using various default emission levels, Monte Carlo (random sampling) simulations were used to randomly combine the various sources of PEMS measurement errors and determine the additive measurement allowance (Buckingham et al., 2009). Although not directly comparable, Table 2 summarises the major errors evaluated in USA and those included in the European approach. It should be reminded also that the importance of these factors is different. For example, in USA time alignment and dynamics are very important (Bougher et al., 2010) as the NTE events can last 30 s, while in EU the effect diminishes because the final result is based on the whole trip (90–120 min).

**Table 2:** Comparison of uncertainties (in EU) (Giechaskiel et al., 2018) and errors (in USA) (Sharp et a, 2009) for determining the conformity factor (in EU) and the measurement allowance (in USA). Note that the percentages refer to different emission levels.

**Measurement Allowance (USA)**	**Value**	**Conformity Factor (EU)**	**Value**
Engine work	1%	Distance	4%
On-road work	6%		
Steady state analyser	5%	Analyser	5%
Steady state EFM	11%	EFM	10%
Transient analyser	2.5%	Time alignment / Dynamics	3%
Transient EFM	0.6%		
Time alignment	1.5%		
EFM pulsations / swirl	1.5%	Boundary conditions	0%
Environmental conditions	3%		
(Drift)	4%	Drift	12–30%
Total (Monte Carlo)	22.5–60%	Total (Error propagation)	24–43%

As a result of the measurement allowance study, a NO_x_ allowance of 600 mg/kWh was adopted for 2007–2009 engines, which was around 23% of the 2007 standard (Sharp et al., 2009, Johnson et al., 2009). The NO_x_ allowance for post 2010 engines was found to be 200 mg/kWh, which was over 60% of the 2010 NTE standard (Sharp et al., 2009). A recent study at 20 mg/kWh levels found 10 mg/kWh measurement error (50%) (Cao et al., 2016).

Table 3 compares the values used in this study with the JRC 2015 study (which assumed linear zero drift) (Weiss et al., 2015), with an on-road evaluation of one 2011 PEMS model with a mobile laboratory using 3 trucks by CE-CERT (Cao et al., 2016), and with a study from KIT (Karlsruher Institut für Technologie) (Appel and Wagner, 2017). KIT conducted a study where a reference PEMS was compared to other PEMS at 4 different European OEMs both in the lab and on the road. Maximum uncertainty estimations were given for the gas analysers, their drift, the EFM and the distance. Other studies with older generation of PEMS, or evaluations at very high emission levels (e.g. Durbin et al., 2007) were not considered in this Table, although the agreement of PEMS and laboratory equipment was good (< 10% differences). Note that the results where two PEMS are compared to each other could theoretically have up to double the estimated measurement uncertainty, in case one is overestimating and the other is underestimating. Typically this uncertainty is expressed with the square root of their sum.

**Table 3:** Uncertainty results (in mg/km, unless otherwise specified) of different studies at 80 mg/km emission levels. n.d. = not determined.

**Component**	**JRC 2015 (**Weiss et al., 2015**)**	**JRC 2017 (**Giechaskiel et al., 2018**)**	**KIT RDE**^a^**(**Appel and Wagner, 2017**)**	**KIT lab (**Appel and Wagner, 2017**)**	**CE-CERT (**Cao et al., 2016**)**
Analyser	6.4 (8%)	4 (5%)	11.4	3.6	n.d.
EFM	3.2 (4%)	8 (10%)	14.6	2.9	< 10% vs. CVS
Drift	16 (20%)	10–15 (5 ppm)	3.8	< 4 ppm	< 1.7 ppm
Worst case drift	16 (20%)	0–10 (5 ppm)	included	–	n.d.
Total	40	20–35	28	< 10	10–15^b^

a The KIT RDE uncertainties are based on the maximum differences of two PEMS, and not compared to reference system (thus they should be divided by a factor of square root of 2, i.e. 1.4 or multiplied by 0.7).

b Based on reported 15% difference at 100 mg/kWh and 50% at 20 mg/kWh and assuming 1 kWh= 1 km.

The uncertainty estimations range from 10 to 40 mg/km (Table 3). The CE-CERT and KIT studies are based on experimental data comparing PEMS with lab grade equipment, while the JRC 2017 study is based on a theoretical basis of uncertainty, where the input values are confirmed by experimental data. The results for the analysers and the EFM are quite close, making the zero drift uncertainty as the main reason of the differences (32 mg/km in the JRC 2015 study, 10–25 mg/km in the JRC 2017 study and < 5 mg/km in the rest studies).

### 6.2 Possibilities for improvement

The uncertainty estimation framework and the assumed values could be refined in subsequent reviews of the uncertainty margins. Our analysis shows that analyser zero drift is a major component of overall PEMS measurement uncertainty. From the 384 zero drift tests, 2.1% were outside the permissible tolerance of 5 ppm. The mean and median values were < 0.5 ppm, indicating that there is no systematic error of the analysers and that the final zero check result is likely due to random variation. If this is true, then the drift will no longer need to be added, but it could be taken into account with the typical uncertainty equations. This assumption, however, needs to be proven with more data: “blank” tests (e.g. checking the zero every few minutes during an on-road test) are necessary. It was also shown that the drift contribution depends on the exhaust flow rate of the vehicle. This means that including the mean exhaust flow rate would give more accurate estimations.

Within the current framework, the reference method (bags from the dilution tunnel) is assumed to have 3% uncertainty. There is a need of experimental data to determine a more accurate value. Instead of subtracting the CVS uncertainty, it could be considered with the error propagation equation in ε_E_.

In the future, the technical specifications pertinent to RDE tests might change. For example, the gas cylinders accuracy could decrease from 2% to 1%, as required in the new type approval laboratory regulation WLTP. Another possibility would be to further reduce the permitted zero drift on the basis of technical improvements of the instruments.

Finally, experimental data might show improvements in the real-world performance of the PEMS. For example, the EFM uncertainty could be proven to be 3% (a value closer to the theoretically expected value) instead of 10% following technological progress in flow measurement.

All these possibilities were not evaluated in the 2017 study due to lack of data. Nevertheless, their effect is at the moment masked by the high contribution to overall uncertainty of the zero drift.

### 6.3 Margin at other emission levels

The uncertainty estimations discussed so far were based on values close to the diesel NO_x_ limits of 80 mg/km. Extending the equation to lower levels is valid only if the uncertainty values remain the same at those lower levels. The validity of this assumption is investigated in this section. From all the components of Table 1, the only ones that could be affected by the lower emissions are the EFM, the NO_x_ analysers and the CVS.

The EFM uncertainty was discussed in Fig. 2. For high flow rates the uncertainty is 2–3%, increasing to 4% for rural conditions (flow rates < 1 m^3^/min) and reaching 10% at idle conditions. However, the EFM uncertainty remains the same for a specific vehicle even if the NO_x_ emission levels decrease.

Fig. 4 showed the NO_x_ analysers measurement uncertainty based on the calibration certificates from 4 PEMS manufacturers. The uncertainty is well within 2% down to approximately 100 ppm, and then gradually increases to approximately 5% at 10 ppm. Based on some experimental NO_x_ real-time data, for emission levels of 80 mg/km, the majority of the NO_x_ spikes are between 60 and 300 ppm. For 20 mg/km, the expected spikes would be between 15 and 75 ppm. In this case, the NO_x_ uncertainty would be on the order of 5% (and not 2%).

At low emission levels the CVS uncertainty also increases. For 80 mg/km emission levels, the NO_x_ concentrations in each bag range from 3 to 9 ppm, thus for 20 mg/km emission levels, 1–2 ppm are expected in the bags. For typical analysers with 20 ppm maximum range, the 2% linearity accuracy applies down to 10% of the full scale (2 ppm). Thus, the bags uncertainty will also be higher at lower emission levels. Consequently, it can be roughly assumed that the relation of the additional PEMS uncertainty (compared to the CVS) at low emission levels remains at the same levels as at the current emission limit of 80 mg/km; in other words, the PEMS and CVS uncertainty increases similarly. This assumption needs to be thoroughly assessed, as it is expected that the uncertainty will be higher for the PEMS. Additionally, the background levels of the instruments have to be considered at these low levels, and this is more difficult (and uncertain) with real time instrument (PEMS).

**Fig. 6:**
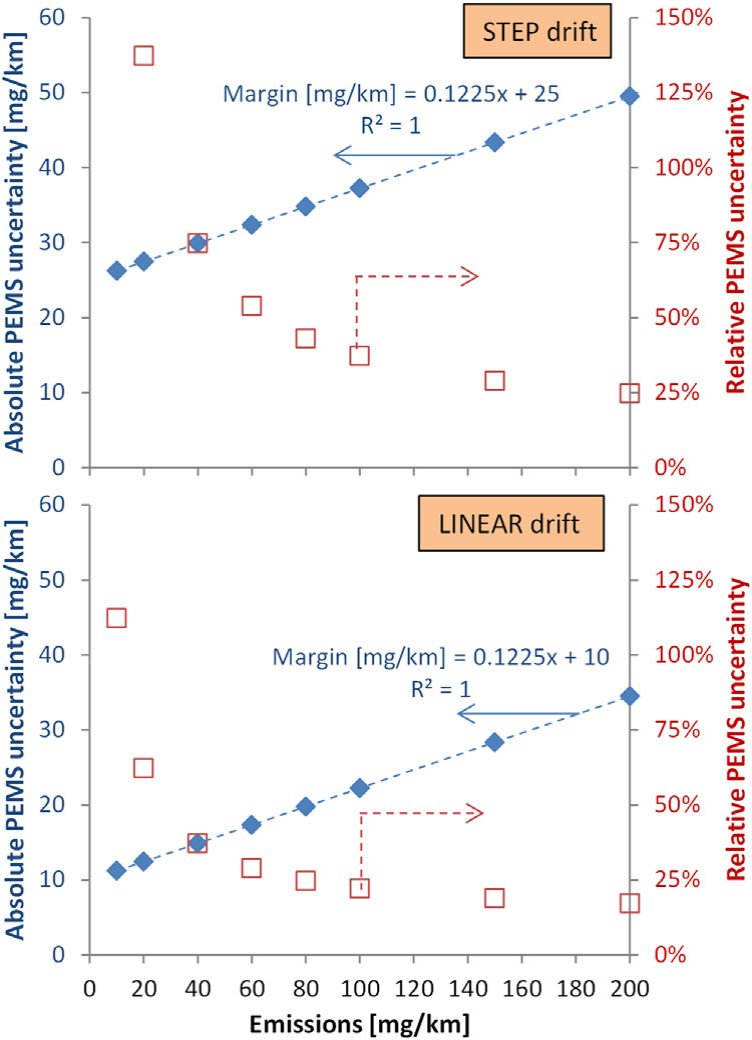
Relative (squares) and absolute (diamonds) uncertainty for different emission levels for linear zero drift (upper panel) and step zero drift (lower panel).

Based on the framework of Fig. 1 and the data of Table 1, and assuming that the uncertainty of PEMS and CVS increase equally as the emission levels decrease, Fig. 6 shows the “additional” PEMS measurement uncertainty. As the emission levels decrease below the current limit value of 80 mg/km, the result of the PEMS will have higher relative uncertainty (expressed in %), but lower absolute uncertainty (expressed in mg/km). For example, for a 60 mg/km emission limit (as is the case of gasoline vehicles), the absolute uncertainty is 17.3–32.3 mg/km (or 29–54% relative uncertainty). At levels around 20 mg/km and lower the linear curve probably breaks down and the values should be treated with caution. However, at low absolute values, uncertainty also becomes less critical with regard to compliance with legal emission limits.

Note also that if the uncertainty of the PEMS equipment for a specific test has to be reported, then the CVS uncertainty should not be subtracted, For example, for 60 mg/km the PEMS measurement uncertainty is 19.1–34.1 mg/km (32–57%). For low emission levels, additionally the higher uncertainty of the analysers has to be considered. For example, for 20 mg/km the PEMS measurement uncertainty is 13.4–28.4 mg/km (instead of 13–28 mg/km) because the NO_x_ analysers accuracy is 5% and not 2%.

For emission levels higher than 80 mg/km the uncertainties of Table 1 should remain constant and the equation given in Fig. 6 should be accurate. The contribution of zero drift becomes less important. For example, at 500 mg/km the (additional to CVS) uncertainty is between 14.2% and 17.2%.

### 6.4 Review procedure in the following years

Based on the experience collected in 2017, subsequent margin reviews should be performed according to the following procedure:

● Collection of new data from all commercially available equipment and/or creation of other data through dedicated experimental campaigns (for Table 1).
● Confirmation with experimental data that the technical requirements are fulfilled both in laboratory and on the road.
● Identification of technical requirements that could be improved in legislation.
● Assessment of each uncertainty according to the framework described in Fig. 1.
● Adjustment of the framework, if necessary (e.g. by the addition of new components, or adjustment of the relation among existing ones).
● Amendment of relevant RDE performance requirements for PEMS equipment and calculation of revised PEMS uncertainty margins.

## 7 Conclusions

With the introduction of the Real-Driving Emissions (RDE) regulation, on-road measurements with Portable Emission Measurement Systems (PEMS) are required as an additional test at type approval. The Not-To Exceed (NTE) limit applicable to RDE tests is based on the current emission standards (Euro 6), and takes into account the additional PEMS measurement uncertainty, but not the variability of measured results related to RDE trip design, vehicle operating conditions, and data evaluation. This additional margin due to instruments measurement uncertainty is currently set to 0.5 but is subject to annual review.

The 2017 annual review set for the first time the framework under which the PEMS uncertainty will be calculated. Data were received from stakeholders to quantify the uncertainty components. One major source of uncertainty was found to be the zero drift. The maximum allowed zero drift of 5 ppm for NO_x_ was simulated as a step drift of 5 ppm at t = 0 and as a linear drift reaching 5 ppm at the end of the test. The simulations of different test cycles with engines of 1.4–3.0 L engine displacement showed an overestimation of the emissions of up to 25 mg/km for the step zero drift, and up to 10 mg/km for the linear zero drift.

Data from one instrument manufacturer showed that exhaust flow meters (EFMs) remained within the regulation requirements (3%) even after one year of use. At low flow rates this uncertainty was around 4%. Other comparisons of EFMs with other EFMs or indirectly determined exhaust flows (e.g. from the dilution tunnel) yielded differences on the order of 10% (even higher in a few cases), but since these other measurements contain uncertainties and are not traceable standards, this 10% is an overestimation of EFM uncertainty.

Based on the proposed framework and the experimentally determined data, a total margin of 0.24–0.43 was calculated for emission level of 80 mg/km, depending on whether the zero drift happens gradually or with a step function at the beginning of the tests. As the emission levels decrease, the result of the PEMS will have higher relative uncertainty, but lower absolute uncertainty. The European Commission based on this study and since there is currently no proof on the real type of zero drift (linear, step) recommended the worst-case margin of 0.43 for its review in 2017.
